# Accuracy of Ultrasound-Estimated Epidural Depth in Transverse Median Versus Parasagittal Oblique Planes for Lower Thoracic Epidural Anesthesia: A Prospective Observational Comparative Study

**DOI:** 10.7759/cureus.114234

**Published:** 2026-08-09

**Authors:** Nitishree Tyagi, Amit K Chauhan, Kriti Bindal, Rohan Bhatia, Shreesh Mehrotra, Harish Koshyari

**Affiliations:** 1 Department of Anaesthesiology, Himalayan Institute of Medical Sciences, Dehradun, IND

**Keywords:** epidural depth, parasagittal oblique plane, thoracic epidural anesthesia, transverse median plane, ultrasonography

## Abstract

Background

Lower thoracic epidural anesthesia is widely used for perioperative analgesia in thoracic and upper abdominal surgeries. However, accurate localization of the epidural space remains technically challenging because of the complex thoracic anatomy. Preprocedural ultrasonography has emerged as a useful adjunct for identifying spinal landmarks and estimating epidural depth before needle insertion. Among the available ultrasound scanning techniques, the transverse median (TM) and parasagittal oblique (PSO) planes are commonly employed, although comparative evidence regarding their accuracy in lower thoracic epidural anesthesia remains limited. This study compared ultrasound-estimated epidural depth with the actual needle depth obtained using the conventional loss-of-resistance (LOR) technique in the TM and PSO planes and evaluated the correlation between body mass index (BMI) and epidural depth measurements.

Methods

This prospective observational comparative study included 70 adult patients (20 to 65 years) with American Society of Anesthesiologists physical status I or II and BMI between 25 and 29.9 kg/m² who underwent elective upper abdominal surgery requiring lower thoracic epidural anesthesia. Patients were equally allocated to the TM group (n=35) and the PSO group (n=35). Preprocedural ultrasound was performed to estimate the skin-to-epidural depth in both planes. Thoracic epidural catheterization was subsequently performed using the conventional LOR technique by an anesthesiologist blinded to the ultrasound measurements. The primary outcome was the accuracy of ultrasound-estimated epidural depth compared with the actual needle depth obtained using the LOR technique. Secondary outcomes included comparisons of epidural depth between the two approaches, correlations with BMI, first-attempt success, needle manipulations, and procedure-related complications.

Results

Baseline demographic and clinical characteristics were comparable between the groups. In the TM group, ultrasound-estimated depth (3.445 ± 0.54 cm) closely matched the actual needle depth (3.449 ± 0.55 cm; p=0.908). Similarly, in the PSO group, ultrasound-estimated depth (4.096 ± 0.66 cm) was comparable to the actual needle depth (4.111 ± 0.61 cm; p=0.524). Both ultrasound-estimated and actual needle depths were significantly greater in the PSO group than in the TM group (p<0.001). Ultrasound-estimated depth showed a very strong positive correlation with actual needle depth in both the TM group (r=0.930, p<0.001) and the PSO group (r=0.977, p<0.001). BMI demonstrated a significant positive correlation only with ultrasound-estimated depth in the TM group (r=0.327, p=0.017), whereas no significant correlations were observed for the remaining comparisons. The PSO group showed a higher first-attempt success rate (94.3% vs. 82.9%) and fewer needle manipulations and complications than the TM group; however, these differences were not statistically significant.

Conclusions

Preprocedural ultrasound accurately estimated epidural depth in both the TM and PSO approaches, showing close approximation to the actual needle depth. While both techniques demonstrated comparable accuracy, the PSO approach demonstrated numerically higher first-attempt success and fewer needle manipulations, although these differences were not statistically significant.

## Introduction

Epidural analgesia is a widely used regional anesthetic technique in which local anesthetic agents, with or without adjuvants, are administered into the epidural space to provide effective analgesia and anesthesia. It may be performed as a single-shot injection or through placement of an indwelling epidural catheter for continuous or intermittent drug administration. Owing to its ability to provide superior perioperative pain control compared with systemic analgesics, epidural analgesia has become an integral component of anesthesia for thoracic, abdominal, obstetric, and lower limb surgeries. In addition to reducing postoperative pain, it decreases opioid consumption and associated adverse effects, thereby facilitating enhanced recovery after surgery [1].

Thoracic epidural analgesia (TEA) is considered the gold standard for perioperative analgesia in thoracic and upper abdominal surgeries. Effective thoracic epidural blockade improves respiratory mechanics, facilitates early mobilization and physiotherapy, attenuates the surgical stress response, and reduces postoperative pulmonary complications [2,3]. By producing sympathetic blockade, TEA also improves myocardial oxygen balance and hemodynamic stability, while its opioid sparing effect minimizes nausea, vomiting, respiratory depression, postoperative ileus, and delayed gastrointestinal recovery [3-5].

Despite these advantages, thoracic epidural catheter placement remains technically demanding. Conventionally, the epidural space is identified using surface anatomical landmarks and the loss-of-resistance (LOR) technique [6,7]. However, the thoracic spine differs considerably from the lumbar region, with long caudally angulated spinous processes, narrow interlaminar spaces, and greater epidural depth. These anatomical characteristics often make needle placement difficult, resulting in multiple attempts, repeated needle redirections, prolonged procedure time, and an increased risk of complications such as accidental dural puncture, vascular puncture, epidural hematoma, neural injury, and failed epidural placement [8,9].

Ultrasonography has emerged as a valuable adjunct for neuraxial anesthesia by allowing visualization of spinal anatomy, identification of vertebral levels, localization of the optimal puncture site, and estimation of the skin-to-epidural space distance before needle insertion [10]. Improvements in ultrasound technology and increasing operator experience have expanded its application in neuraxial procedures. Preprocedural ultrasound has been shown to improve first-attempt success, reduce the number of needle passes, and increase procedural safety, particularly in patients with obesity, spinal deformity, or poorly palpable anatomical landmarks [11]. Although ultrasound guidance is well established for lumbar neuraxial procedures, its application in thoracic epidural anesthesia remains relatively limited because of the narrower acoustic window and the complex thoracic anatomy [12].

Two commonly used ultrasound scanning approaches for thoracic epidural imaging are the transverse median (TM) and parasagittal oblique (PSO) planes. The TM approach visualizes the spinous processes and interspinous spaces, whereas the PSO approach provides an oblique view of the laminae and interlaminar spaces and may offer a wider acoustic window with improved visualization of the ligamentum flavum dura mater complex [8]. Accurate estimation of skin-to-epidural depth is essential for safe needle advancement and successful epidural catheter placement. However, comparative evidence regarding the accuracy of epidural depth estimation using the TM and PSO approaches in the thoracic region remains limited [9,10]. Therefore, the present study primarily aimed to evaluate the accuracy of ultrasound-estimated epidural depth by comparing it with the actual needle depth obtained using the conventional LOR technique in the TM and PSO planes. Secondary objectives included comparison of procedural outcomes and evaluation of the relationship between BMI and epidural depth measurements.

## Materials and methods

Study design and ethical approval

This prospective observational comparative study was conducted at a tertiary care teaching hospital after obtaining approval from the Institutional Ethics Committee (Ref. No. SRHU/HIMS/ETHICS/2024/353 dated 19/10/24). The study was conducted over a period of one year, from 01 January 2025 to 31 December 2025. Written informed consent was obtained from all participants before enrolment, and the study was conducted in accordance with the ethical principles of the Declaration of Helsinki. The reporting of this study follows the Strengthening the Reporting of Observational Studies in Epidemiology (STROBE) guidelines (Appendix 1).

Study population

A total of 70 adult patients aged 20 to 65 years with American Society of Anesthesiologists (ASA) physical status I or II and a body mass index (BMI) between 25 and 29.9 kg/m², scheduled to undergo elective upper abdominal surgery requiring lower thoracic epidural anesthesia, were enrolled. Patients who refused epidural anesthesia, had a history of neurological disorders, previous spinal surgery, spinal deformity, local infection at the puncture site, coagulopathy, or any contraindication to neuraxial anesthesia were excluded. During the preanesthetic evaluation, demographic characteristics, including age, sex, height, weight, BMI, and ASA physical status, were recorded.

Preprocedural preparation

All patients adhered to standard fasting guidelines, including six hours for solid food and two hours for clear fluids. In the operating room, intravenous access was secured, and routine monitoring was instituted, including electrocardiography, systolic blood pressure, diastolic blood pressure, mean arterial pressure, peripheral oxygen saturation, and temperature. Patients were positioned in the sitting posture, and the thoracic region was prepared with 6% chlorhexidine solution under strict aseptic precautions before sterile draping. The T9-T10, T10-T11, and T11-T12 intervertebral spaces were initially identified using surface anatomical landmarks.

Ultrasound assessment

**Figure 1:**
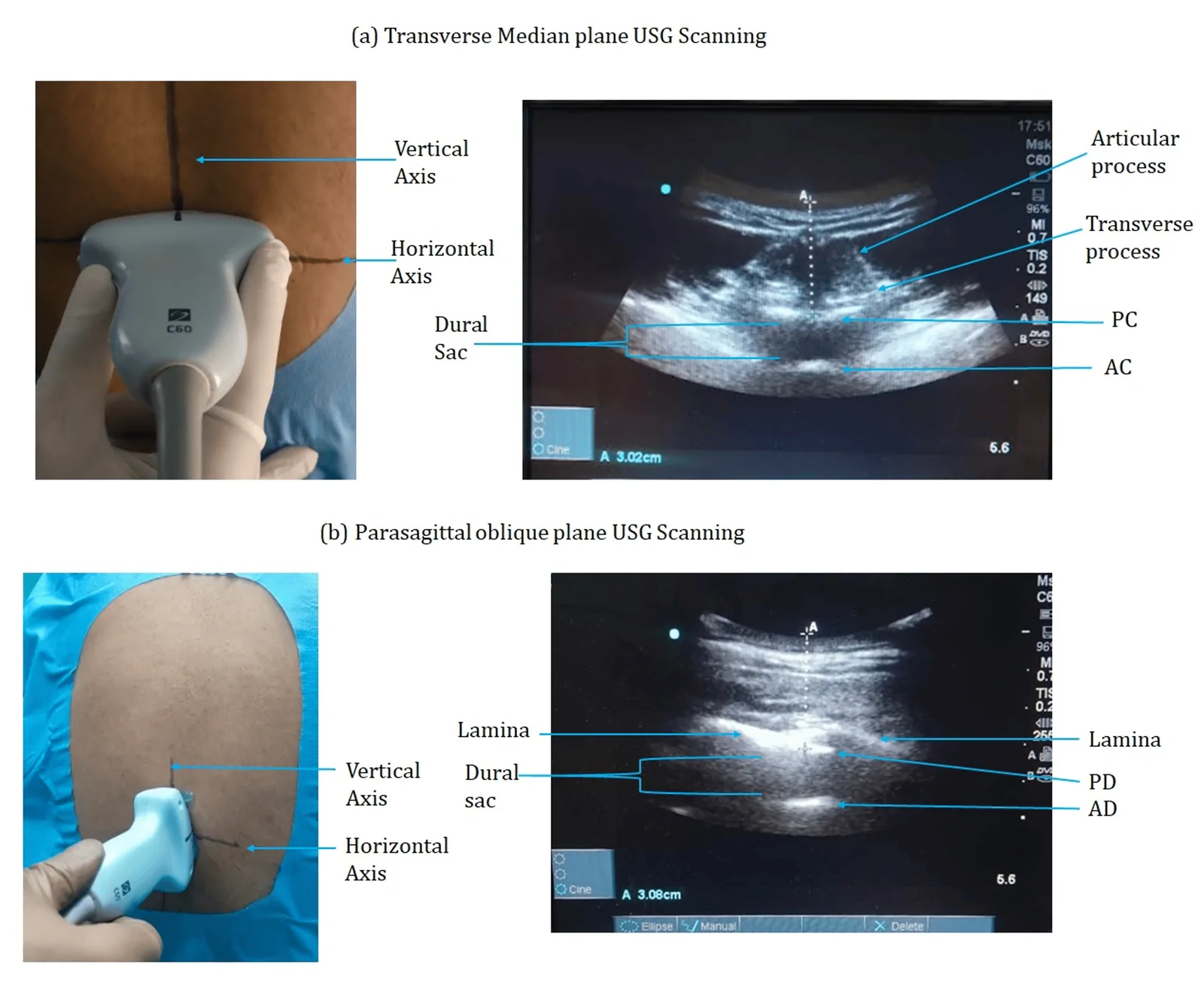
Representative ultrasound images of the TM and PSO scanning planes used for lower thoracic epidural anesthesia. Panel (a) shows the TM view with the probe position and corresponding ultrasound image demonstrating the transverse process, posterior complex, anterior complex, dural sac, and ultrasound-estimated epidural depth. Panel (b) shows the PSO view with the probe position and corresponding ultrasound image demonstrating the lamina, dural sac, posterior complex, anterior complex, and ultrasound-estimated epidural depth. AC, anterior complex; AD, anterior dura; PC, posterior complex; PD, posterior dura; PSO, parasagittal oblique; TM, transverse median; USG, ultrasonography.

Preprocedural ultrasound examination was performed by an experienced anesthesiologist using a portable ultrasound system (SonoSite M Turbo, Fujifilm SonoSite Inc., Bothell, WA, USA) equipped with a 2-5 MHz curvilinear transducer. Probe orientation and image acquisition were standardized for all patients. Minimal probe pressure was applied during scanning to minimize tissue compression while measuring epidural depth. Ultrasound scanning was performed in both the TM and PSO planes (Figures 1A, 1B, respectively). In the TM view (Figure 1A), the spinous process appeared as a hyperechoic structure with posterior acoustic shadowing, while the interlaminar space provided an acoustic window for visualization of the posterior complex (PC), representing the ligamentum flavum dura mater complex, and the anterior complex, representing the posterior longitudinal ligament vertebral body complex. The optimal TM image demonstrated the characteristic hyperechoic "=" sign, whereas the PSO view (Figure 1B) demonstrated the characteristic "camel hump" appearance. The PC served as the reference landmark for epidural depth estimation. After obtaining the optimal sonographic image, skin markings corresponding to the probe orientation were made, and the needle insertion site was identified at the intersection of these markings. Ultrasound-estimated epidural depth was measured from the skin to the PC using the built-in electronic calipers. Representative ultrasound images with labeled anatomical landmarks are shown in Figure 1.

Epidural catheter placement

Thoracic epidural placement was subsequently performed by a second experienced anesthesiologist with expertise in thoracic epidural catheter placement and preprocedural neuraxial ultrasonography who was blinded to the ultrasound-estimated depth measurements. An 18-gauge Tuohy epidural needle was inserted at the predetermined puncture site following the ultrasound beam trajectory. Minor cephalad or caudal adjustments were made whenever necessary to achieve optimal needle advancement. The epidural space was identified using the conventional LOR technique with air or saline according to operator preference. Following successful localization of the epidural space, the actual needle depth was measured from the skin entry point to the corresponding needle marking using a sterile linear scale.

Outcome measures

The primary outcome was the accuracy of ultrasound-estimated epidural depth compared with the actual needle depth. Secondary outcomes included comparison of epidural depth between the two ultrasound approaches, correlation of ultrasound-estimated depth and needle depth with BMI, first-attempt success rate, need for needle redirection or reangulation, change of intervertebral level, and procedure-related complications, including failed block, dural puncture, post dural puncture headache, and vascular puncture.

Sample size calculation

The sample size was determined with reference to the pilot study by Khemka et al. [13], which demonstrated a very strong correlation between ultrasound-estimated epidural depth and actual needle depth in both the TM and PSO planes (Pearson correlation coefficient = 0.99 in both groups). Assuming a two-sided significance level of 5%, a study power of 90%, and allowing for adequate precision in estimating the accuracy of ultrasound-estimated depth compared with actual needle depth, a minimum of 30 participants per group was considered sufficient. To compensate for possible dropouts and incomplete data, 35 patients were enrolled in each group, resulting in a total sample size of 70 participants. Patients were allocated consecutively into the TM and PSO groups until the required sample size was achieved.

Statistical analysis

Statistical analysis was performed using IBM SPSS Statistics for Windows, Version 27.0 (IBM Corp., Armonk, NY, USA). Continuous variables are presented as mean ± standard deviation (SD), while categorical variables are expressed as frequencies and percentages. Data normality was assessed before statistical analysis. Paired Student's t test was used to compare ultrasound-estimated depth and actual needle depth within each group, whereas independent Student's t test was used for comparisons between groups. Pearson correlation analysis was performed to evaluate the relationship between ultrasound-estimated epidural depth and actual needle depth within each group. Pearson correlation analysis was also used to assess the relationship between BMI and epidural depth measurements. Categorical variables were analyzed using the Chi square test or Fisher's exact test, as appropriate. A two-sided p value of less than 0.05 was considered statistically significant.

## Results

A total of 70 patients were enrolled and equally allocated to the TM group (n=35) and the PSO group (n=35). The mean age of patients was comparable between the TM and PSO groups (48.51 ± 9.40 vs. 49.60 ± 8.52 years, p=0.614). Similarly, there were no significant differences in mean weight (70.71 ± 8.26 vs. 71.74 ± 6.32 kg, p=0.560), mean height (161.44 ± 8.46 vs. 162.46 ± 7.60 cm, p=0.598), or mean BMI (27.05 ± 1.12 vs. 27.17 ± 1.36 kg/m², p=0.693) between the two groups. The distribution of sex was also comparable, with females comprising 19 (54.3%) patients in the TM group and 20 (57.1%) in the PSO group, while males accounted for 16 (45.7%) and 15 (42.9%) patients, respectively (p=0.810). Most participants belonged to ASA physical status II, accounting for 31 (88.6%) patients in the TM group and 34 (97.1%) in the PSO group, whereas ASA I patients constituted 4 (11.4%) and 1 (2.9%) patients, respectively (p=0.164). Overall, there were no statistically significant differences in baseline demographic or clinical characteristics between the two groups, indicating that they were comparable at baseline (Table 1).

**Table 1:** Comparison of demographic and clinical characteristics between the TM and PSO groups Values are presented as mean ± SD or n (%). ASA, American Society of Anesthesiologists; PSO, parasagittal oblique; SD, standard deviation; TM, transverse median; χ², Chi-square.

Variable	Category	TM group (n=35)	PSO group (n=35)	Test value	p‑value
Age (years)	–	48.51 ± 9.40	49.60 ± 8.52	t=0.506	0.614
Weight (kg)	–	70.71 ± 8.26	71.74 ± 6.32	t=0.585	0.560
Height (cm)	–	161.44 ± 8.46	162.46 ± 7.60	t=0.529	0.598
Body mass index (kg/m²)	–	27.05 ± 1.12	27.17 ± 1.36	t=0.397	0.693
Sex	Female	19 (54.3%)	20 (57.1%)	χ²=0.058	0.810
	Male	16 (45.7%)	15 (42.9%)		
ASA status	ASA I	4 (11.4%)	1 (2.9%)	χ²=1.938	0.164
	ASA II	31 (88.6%)	34 (97.1%)		

The mean ultrasound-estimated epidural depth in the TM group was 3.445 ± 0.54 cm, while the mean actual needle depth was 3.449 ± 0.55 cm. The difference between the two measurements was not statistically significant (paired t=0.116, p=0.908). Similarly, in the PSO group, the mean ultrasound-estimated depth was 4.096 ± 0.66 cm, whereas the mean actual needle depth was 4.111 ± 0.61 cm, with no statistically significant difference between the measurements (paired t=0.644, p=0.524). These findings demonstrate that ultrasound-estimated epidural depth closely approximated the actual needle depth in both the TM and PSO groups (Table 2).

**Table 2:** Comparison of ultrasound-estimated depth and actual needle depth in the TM and PSO groups PSO, parasagittal oblique; SD, standard deviation; TM, transverse median.

Group	Variable	Mean ± SD	t-test	p-value
TM group (n=35)	Ultrasound depth (cm)	3.445 ± 0.54	0.116	0.908
	Needle depth (cm)	3.449 ± 0.55		
PSO group (n=35)	Ultrasound depth (cm)	4.096 ± 0.66	0.644	0.524
	Needle depth (cm)	4.111 ± 0.61		

**Figure 2:**
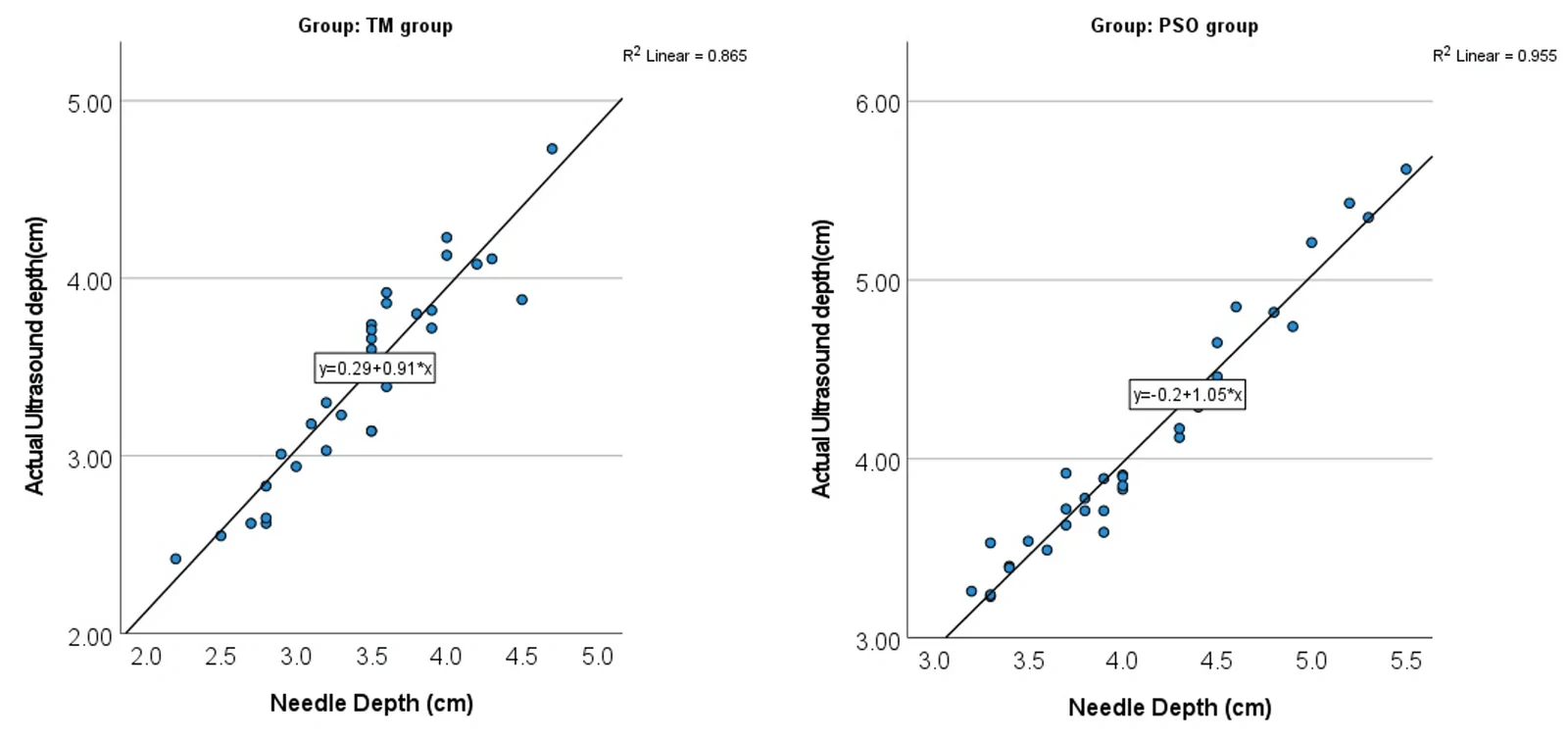
Correlation between ultrasound-estimated depth and actual needle depth PSO, parasagittal oblique; TM, transverse median.

Pearson correlation analysis demonstrated a very strong positive correlation between ultrasound-estimated epidural depth and actual needle depth in both groups. In the TM group, ultrasound-estimated depth was highly correlated with actual needle depth (r=0.930, p<0.001). Similarly, the PSO group showed an even stronger positive correlation between ultrasound-estimated depth and actual needle depth (r=0.977, p<0.001). These findings indicate that preprocedural ultrasound accurately predicted the actual needle depth in both approaches (Figure 2).

Correlation analysis demonstrated a significant positive correlation between BMI and ultrasound-estimated epidural depth in the TM group (r=0.327, p=0.017), indicating that higher BMI was associated with greater ultrasound-estimated epidural depth. However, no significant correlation was observed between BMI and actual needle depth in the TM group (r=0.081, p=0.644). In the PSO group, neither the correlation between BMI and ultrasound-estimated epidural depth (r=0.248, p=0.186) nor the correlation between BMI and actual needle depth (r=0.268, p=0.119) reached statistical significance. Overall, BMI showed a significant association only with ultrasound-estimated epidural depth in the TM group, whereas no significant relationship was observed with actual needle depth or with either depth measurement in the PSO group (Table 3).

**Table 3:** Correlation between body mass index and epidural depth measurements in the TM and PSO groups PSO, parasagittal oblique; TM, transverse median.

Group	Variables	Pearson correlation (r)	p-value
TM group (n=35)	BMI vs. ultrasound depth	0.327	0.017
	BMI vs. needle depth	0.081	0.644
PSO group (n=35)	BMI vs. ultrasound depth	0.248	0.186
	BMI vs. needle depth	0.268	0.119

Comparison of epidural depth measurements between the two groups demonstrated that the mean ultrasound-estimated depth was significantly greater in the PSO group than in the TM group (4.096 ± 0.66 cm vs. 3.445 ± 0.54 cm; t=4.538, p<0.001). Similarly, the mean actual needle depth was significantly higher in the PSO group (4.111 ± 0.61 cm) compared with the TM group (3.449 ± 0.55 cm; t=4.763, p<0.001). These findings indicate that both the ultrasound-estimated epidural depth and the actual needle depth were significantly greater in the PSO group than in the TM group (Table 4).

**Table 4:** Comparison of ultrasound-estimated depth and actual needle depth between the TM and PSO groups PSO, parasagittal oblique; SD, standard deviation; TM, transverse median.

Variable	TM group (n=35), mean ± SD	PSO group (n=35), mean ± SD	t-value	p-value
Ultrasound depth (cm)	3.445 ± 0.54	4.096 ± 0.66	4.538	<0.001
Needle depth (cm)	3.449 ± 0.55	4.111 ± 0.61	4.763	<0.001

The first-attempt success rate was higher in the PSO group, with successful epidural placement achieved in 33 (94.3%) patients, compared with 29 (82.9%) patients in the TM group. However, the difference in the number of attempts required for successful epidural placement was not statistically significant (p=0.232). Needle reangulation was required in 6 (17.1%) patients in the TM group and 2 (5.7%) patients in the PSO group (p=0.259). Similarly, cephalad needle redirection was performed in 4 (11.4%) patients in the TM group and 2 (5.7%) patients in the PSO group (p=0.673), while caudal needle redirection was required in 2 (5.7%) patients in the TM group and in none of the patients in the PSO group (p=0.493). A change in the intervertebral space was required in 2 (5.7%) patients in the TM group but in none of the patients in the PSO group (p=0.493). Regarding procedural complications, failed epidural block occurred in 2 (5.7%) patients in the TM group and in none of the patients in the PSO group (p=0.493). Dural puncture and post-dural puncture headache each occurred in 2 (5.7%) patients in the TM group, whereas no cases were observed in the PSO group (p=0.614 for both). No hemorrhage (blood tap) was reported in either group. Overall, although procedural success tended to be higher and complications were fewer in the PSO group, none of these differences reached statistical significance (Table 5).

**Table 5:** Comparison of procedural outcomes and adverse events between the TM and PSO groups PSO, parasagittal oblique; TM, transverse median.

Variable	TM group (n=35)	PSO group (n=35)	Test value	p-value
1 attempt	29 (82.9%)	33 (94.3%)	χ²=2.925	0.232
2 attempts	4 (11.4%)	2 (5.7%)		
>2 attempts	2 (5.7%)	0 (0.0%)		
Needle reangulation	6 (17.1%)	2 (5.7%)	χ²=2.258	0.259
Cephalad needle redirection	4 (11.4%)	2 (5.7%)	χ²=0.729	0.673
Caudal needle redirection	2 (5.7%)	0 (0.0%)	χ²=2.059	0.493
Need for change of intervertebral space	2 (5.7%)	0 (0.0%)	χ²=2.059	0.493
Failed block	2 (5.7%)	0 (0.0%)	χ²=2.059	0.493
Hemorrhage (blood tap)	0 (0.0%)	0 (0.0%)	–	–
Dural puncture	2 (5.7%)	0 (0.0%)	χ²=1.061	0.614
Post-dural puncture headache	2 (5.7%)	0 (0.0%)	χ²=1.061	0.614

## Discussion

The present study evaluated the accuracy of ultrasound-estimated epidural depth by comparing it with the actual needle depth obtained using the conventional LOR technique in the TM and PSO planes during lower thoracic epidural anesthesia. In addition, the study assessed the correlation between BMI and epidural depth measurements in both approaches. Baseline demographic and clinical characteristics were comparable between the two groups, minimizing potential confounding and allowing an unbiased comparison of the two ultrasound scanning planes.

The principal finding of the present study was that ultrasound-estimated epidural depth closely approximated the actual needle depth in both the TM and PSO groups. This observation was further supported by a very strong positive correlation between ultrasound-estimated depth and actual needle depth in both groups (TM: r=0.930; PSO: r=0.977; both p<0.001). These findings indicate that preprocedural ultrasound provides a reliable estimate of epidural depth before needle insertion and directly support the primary objective of the study.

No statistically significant difference was observed between ultrasound-estimated depth and needle depth within either group. These findings confirm that preprocedural ultrasound is an accurate and reliable method for estimating epidural depth during lower thoracic epidural placement. Similar findings were reported by Khemka et al., who demonstrated close correspondence between ultrasound-estimated and actual needle depth in both the TM and PSO planes, supporting the use of ultrasound as a dependable adjunct for thoracic epidural catheter placement [13].

Although both ultrasound approaches accurately predicted epidural depth, the mean ultrasound-estimated depth and actual needle depth were significantly greater in the PSO group than in the TM group. This difference is most likely explained by the longer tissue trajectory associated with the paramedian approach rather than reduced measurement accuracy. Therefore, the greater measured epidural depth in the PSO group should not be interpreted as superior accuracy of the PSO approach. Similar observations have been reported in a previous study comparing the two scanning planes, indicating that the PSO approach measures a greater skin-to-epidural distance because of its oblique needle pathway while maintaining comparable accuracy [13].

With respect to procedural performance, the majority of epidural catheter placements were successfully achieved on the first attempt in both groups. Although the difference was not statistically significant, the PSO group demonstrated a higher first-attempt success rate and fewer needle reangulations, needle redirections, and changes of intervertebral space than the TM group. These findings indicate a trend toward easier epidural placement with the PSO approach; however, the differences were not statistically significant and should be interpreted cautiously. Avramescu et al. demonstrated that the PSO plane provides superior visualization of thoracic neuraxial structures because the wider acoustic window minimizes acoustic shadowing from the spinous processes, thereby improving identification of the interlaminar space [14]. Similarly, Mudavath et al. reported a significantly higher first-attempt success rate and fewer needle attempts using the paramedian approach compared with the median approach during thoracic epidural placement, while the incidence of complications remained comparable between the groups. Their findings support the trend toward improved procedural ease observed in the present study [15]. Abd Elhady et al. also reported that ultrasound guidance reduced needle attempts and redirections compared with conventional landmark-based techniques, emphasizing the value of ultrasound-assisted neuraxial procedures [16].

Correlation analysis demonstrated a weak but statistically significant positive correlation between BMI and ultrasound-estimated epidural depth in the TM group, whereas no significant correlation was observed between BMI and actual needle depth, or between BMI and either depth measurement, in the PSO group. Since only overweight patients with a BMI between 25 and 29.9 kg/m² were included, the influence of obesity on epidural depth could not be adequately assessed. These findings should be interpreted cautiously because the observed association was weak and was not consistently demonstrated across both approaches. Within this moderate BMI range, preprocedural ultrasound maintained good predictive accuracy for thoracic epidural depth estimation.

The overall incidence of procedure-related complications was low in both groups. Although failed epidural block, dural puncture, and post-dural puncture headache occurred only in the TM group, the differences were not statistically significant. Previous studies have shown that minimizing needle attempts and redirections reduces the likelihood of neuraxial complications. Hermanides et al. emphasized that repeated needle manipulations increase the risk of complications, highlighting the importance of accurate epidural depth estimation and optimal needle trajectory planning [17]. The lower frequency of needle manipulations observed in the PSO group may therefore have potential clinical relevance despite the lack of statistical significance.

This study has several limitations. First, it was conducted at a single center with a relatively small sample size, which may limit the generalizability of the findings. Second, only patients with a BMI between 25 and 29.9 kg/m² and ASA physical status I or II were included; therefore, the results may not be applicable to patients with obesity, spinal deformities, or other high-risk populations. Third, ultrasound examinations were performed by experienced anesthesiologists, and the findings may differ with less experienced operators. Finally, actual needle depth was measured using the LOR technique, which remains operator dependent despite being the current clinical standard.

The findings of the present study support the use of preprocedural ultrasound as a valuable adjunct for lower thoracic epidural placement. Both the TM and PSO approaches accurately estimated epidural depth and closely approximated the actual needle depth. Although the PSO approach demonstrated numerically higher first-attempt success and fewer needle manipulations, these differences were not statistically significant and should be interpreted cautiously. Therefore, both approaches appear to be reliable for lower thoracic epidural catheter placement, with the PSO approach showing a possible procedural advantage that requires confirmation in larger studies. The study was powered for the primary outcome and may have been underpowered to detect differences in secondary outcomes such as first-attempt success and procedure-related complications. In addition, measurement of actual needle depth using the LOR technique may have been influenced by needle angulation and operator technique. Furthermore, the use of either air or saline for the LOR technique reflected routine clinical practice and may have introduced minor procedural variability.

## Conclusions

Preprocedural ultrasonography accurately estimated epidural depth in both the TM and PSO, demonstrating close approximation to the actual needle depth obtained using the conventional LOR technique during lower thoracic epidural anesthesia. Although both approaches provided comparable accuracy, the PSO approach demonstrated numerically higher first-attempt success and fewer needle manipulations; however, these differences were not statistically significant. Ultrasound assessment may therefore improve procedural planning and enhance the safety of thoracic epidural placement. Larger multicenter studies, including patients with a wider range of BMI, are warranted to validate these findings.

## Appendices

Appendix 1 

**Table 6:** STROBE checklist

Item no.	Recommendation	Location in manuscript
1a	Indicate the study's design with a commonly used term in the title or abstract	Yes (Title and Abstract)
1b	Provide an informative and balanced summary	Yes (Abstract)
2	Explain the scientific background and rationale	Yes (Introduction)
3	State specific objectives, including any prespecified hypotheses	Yes (Introduction)
4	Present key elements of study design early in the paper	Yes (Materials and Methods)
5	Describe the setting, locations, and relevant dates	Yes (Study Design and Ethical Approval)
6a	Eligibility criteria and participant selection	Yes (Study Population)
6b	Matching criteria (cohort only, if applicable)	N/A
7	Clearly define outcomes, exposures, predictors, confounders	Yes (Outcome Measures)
8	Data sources and methods of assessment	Yes (Ultrasound Assessment; Epidural Catheter Placement)
9	Describe efforts to address bias	Yes (Blinding; Discussion)
10	Explain study size	Yes (Sample Size Calculation)
11	Explain quantitative variables	Yes (Statistical Analysis)
12a	Describe statistical methods	Yes (Statistical Analysis)
12b	Methods for subgroups/interactions	N/A
12c	Explain missing data	N/A (No missing data)
12d	Loss to follow-up	N/A
12e	Sensitivity analyses	N/A
13a	Numbers of participants at each stage	Yes (Results)
13b	Reasons for non-participation	N/A
13c	Flow diagram	N/A
14a	Participant characteristics	Yes (Results, Table 1)
14b	Missing data	N/A (No missing data)
14c	Follow-up time	N/A
15	Outcome data	Yes (Results, Tables 2–5)
16a	Main results	Yes (Results)
16b	Category boundaries	Yes (Study Population: BMI categories)
16c	Absolute risk	N/A
17	Other analyses	Yes (Correlation analyses)
18	Summarize key results	Yes (Discussion)
19	Discuss limitations	Yes (Discussion)
20	Interpretation	Yes (Discussion and Conclusion)
21	Generalizability	Yes (Discussion)
22	Funding	Yes (Acknowledgments/Funding statement)
