# Correction: Accuracy of Ultrasound-Estimated Epidural Depth in Transverse Median Versus Parasagittal Oblique Planes for Lower Thoracic Epidural Anesthesia: A Prospective Observational Comparative Study

**DOI:** 10.7759/cureus.c488

**Published:** 2026-10-09

**Authors:** Nitishree Tyagi, Amit K Chauhan, Kriti Bindal, Rohan Bhatia, Shreesh Mehrotra, Harish Koshyari

**Affiliations:** 1 Department of Anaesthesiology, Himalayan Institute of Medical Sciences, Dehradun, IND

The listed author order has been revised at the request of the authors. Dr. Kriti Bindal is now listed as the third author, with Dr. Rohan Bhatia listed as the fourth author. All listed authors consented to this change.

